# The discovery of hydrogen bonds in DNA and a re-evaluation of the 1948 Creeth two-chain model for its structure

**DOI:** 10.1042/BST20180158

**Published:** 2018-09-06

**Authors:** Stephen E. Harding, Guy Channell, Mary K. Phillips-Jones

**Affiliations:** 1 National Centre for Macromolecular Hydrodynamics, School of Biosciences, University of Nottingham, Sutton Bonington LE12 5RD, U.K.; 2 Kulturhistorisk Museum, Universitetet i Oslo, Postboks 6762, St. Olavs plass, 0130 Oslo, Norway

**Keywords:** biological models, DNA, hydrodynamics

## Abstract

We recall the experimental approaches involved in the discovery of hydrogen bonds in deoxyribonucleic acid (DNA) made 70 years ago by a team of scientists at University College Nottingham led by J.M. Gulland, and in relation to previous studies. This discovery proved an important step in the elucidation of the correct structure for DNA made by J.D. Watson and F.H.C. Crick, as acknowledged in ‘*The Double Helix*’. At that time of the discovery, however, it was impossible to delineate between inter- and intra-chain hydrogen bonds. We also consider in the light of more recent hydrodynamic theory a tentative model for DNA proposed by Gulland's and D.O. Jordan's PhD student J.M. Creeth in his PhD thesis of 1948, with the correct prediction of two chains with a sugar-phosphate backbone on the exterior and hydrogen-bonded bases between the nucleotide bases of opposite chains in the interior. Our analysis shows that his incorporation of alternating breaks in the two-chain structure was not necessary to explain the viscosity data on scission of hydrogen bonds after titrating to high or low pH. Although Creeth's model is a depiction of DNA structure alone, he could not know whether the hydrogen bonding was intermolecular, although this was subsequently proved correct by others. The mechanisms by which replicative processes occurred were of course unknown at that time, and so, he could not have realised how closely his tentative model resembled steps in some viral replicative mechanisms involving the molecule of life that he was working on.

## Introduction

The elucidation of the double-helix structure for DNA was one of the most exciting and momentous discoveries of the 20th century. The correct model brilliantly constructed by J.D. Watson — a postdoctoral Research Fellow — and F.H.C. Crick — a PhD student — with two sugar-phosphate chains running antiparallel ‘space group C2’ to each other and with paired pyrimidine–purine nucleotide bases held together by hydrogen bonds was, as acknowledged by Watson and Crick, dependent on the data of others [1].

In this short article, written to commemorate the 70th anniversary of the discovery of hydrogen bonds in DNA by scientists at the University of Nottingham and part of the meeting held on 10 November 2017 to commemorate this [2], we review the experiments undertaken on highly purified calf-thymus DNA that led to the hydrogen bond discovery communicated in three papers published in the *Journal of the Chemical Society*, with the definitive study based on hydrodynamic experiments by J.M. Creeth, J.M. Gulland and D.O. Jordan [3,4]. This discovery also built on the contributions of other groups, most notably from Stockholm, Uppsala and Oxford. Reference has been made previously to the discovery by the Nottingham team, but this has generally focused on the contributions of Gulland [5], or Jordan and Gulland [6,7]. The discovery is acknowledged in the latest version of J.D. Watson's classical book *The Double Helix* [8], including the important role played by Creeth. We take a fresh look at the hydrogen bond discovery by the Nottingham team in the light of contributions from scientists elsewhere. Furthermore, in his PhD thesis — now fully available online [4] — Creeth proposed a two-chain model for DNA that appears to be not too dissimilar from the final and correct structure worked out by Watson and Crick some 5 years later. The existence of this model was made aware to one of us (S.E.H.) — a former postdoctoral Research Fellow — during a tea-room conversation with him in the Department of Medicine laboratories at the University of Bristol in 1982. ‘Tucked away in the corner of my thesis was a structure which proved not too dissimilar from the final structure worked out at Cambridge’.

In this study, we take a look at the hydrogen bond discovery by the Nottingham team and how it built on the contributions from scientists elsewhere. We also re-examine in the light of modern hydrodynamic theory the proposed structure of Creeth [4] with its correct prediction of two chains with the sugar-phosphate backbone on the exterior and hydrogen bonds between the nucleotide bases of opposite chains in the interior, and consider how close he came — but how far he was — from the final structure worked out by Watson and Crick. Using more recently developed hydrodynamic theory, we now show that his incorporation of alternating breaks in his two-chain structure was not necessary to explain the viscosity data on scission of the hydrogen bonds on titrating to high or low pH.

## State of knowledge up to 1947

Prior to 1947, there had been growing interest in DNA (and indeed RNA) as it was considered this might be a substance which was associated with genes or inheritance [9]. Debate of the role of DNA in genes and heredity began in 1944 with the identification of DNA as the ‘transforming principle’ in pneumococci (see ref. [10]). Furthermore, the first proposal that macromolecular DNA was related to macromolecular microsomal (ribosomal) RNA, which in turn produces cytoplasmic enzymes, was made in 1947 by Boivin and Vendrely [11]. However, during the 1940s, there remained a core of researchers who retained the view that it was the protein component of chromosomes, with its 20 or so amino acids (which could potentially offer the required wide variation in combinations), that was still likely to turn out to be the mechanism of heredity [12,13]. This idea and the ‘tetranucleotide’ theory that underpinned it were not finally overturned in favour of nucleic acid until 1950 when Erwin Chargaff reported that the four bases in DNA could be present in varying amounts depending on the species from which the DNA was derived [14].

The DNA molecule was known to be polymeric with a high molecular mass [15,16]. It was also known to consist of polymeric deoxyribose linked together in unbranched chains by phospho-diester bonds — as opposed to glycosidic bonds found in other polysaccharides and glycoconjugates — together with pyrimidine ‘bases’ namely thymine (T) and cytosine (C), and the purine bases adenine (A) and guanine (G). Working on highly purified calf-thymus DNA, Signer et al. [16] showed that these molecules had a molecular mass (molar mass) of between 0.5 and 1.0 MDa. Furthermore, measurements on the optical anisotropy of solutions had suggested that the pyrimidine and purine bases were situated in planes perpendicular to the longitudinal axis of the DNA molecule [16]. This was reinforced by X-ray diffraction studies by Astbury and Bell [17] and Bell [18] at Leeds, who determined the spacing between the bases to be ∼0.34 nm.

## Step one: non-degraded high-purity DNA

By 1947, a team of scientists had been assembled in the Nucleic Acid Laboratories in the Department of Chemistry at what was then University College Nottingham. The team, under the leadership of Professor J. Masson Gulland FRS, included Dr D.O. ‘Doj’ Jordan — a lecturer in Physical Chemistry — and three PhD students — Cedric J. Threlfall, H.F.W. Taylor and J. Michael Creeth — who together published a series of three papers in the *Journal of the Chemical Society*, culminating in the unequivocal demonstration of the existence of intra- and/or intermolecular hydrogen bonds between the nucleotide bases in a DNA molecule.

Of utmost importance when performing structural evaluations on nucleic acids is the molecular integrity of the material being studied [19]. So, the first paper, by Gulland et al. [20], focused on the production of DNA from calf thymus of the highest degree of integrity and purity. This involved avoidance of the application of high acid or high alkali in the extraction process. A mild extraction procedure was therefore employed in which only neutral solutions and temperatures near 0°C were used, improving on an earlier extraction procedure by Bang [21] and later by Hammersten in Stockholm [22], to give a product that was believed to be protein-free. This was based on (i) negative Biuret and Sakaguchi tests and (ii) total nitrogen determinations matching closely only the calculated nitrogenous base content [23]. It was particularly important to ensure (and verify) removal of protein (as Gulland presumably recognised), as it was still believed by some at that time (including A. Mirsky) to be the basis of heredity; it was suggested that protein could still be present, albeit in low amounts, but remain undetected due to insensitive protein determination methods.

**Figure 1.**
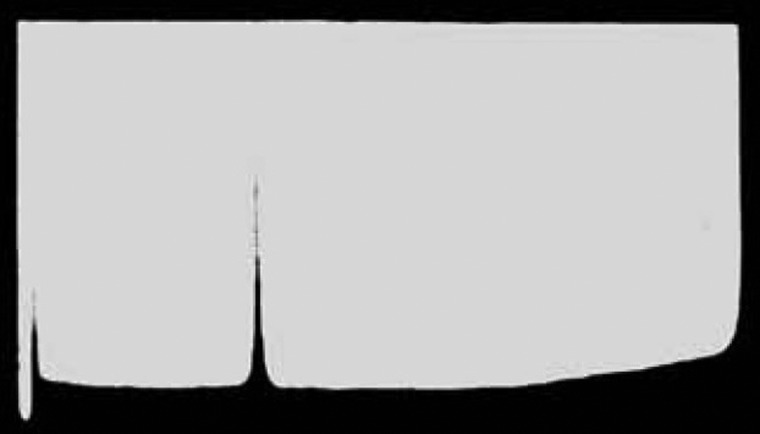
Schlieren profile from a Svedberg analytical ultracentrifuge showing a single hyper-sharp boundary for the preparation of calf-thymus DNA of high purity prepared by Gulland et al. [20] in 0.2 M NaCl. The direction of sedimentation is from left to right. Adapted from Cecil and Ogston [24]. The peak to the left is the air/solvent meniscus. Reproduced with permission from the Royal Society of Chemistry.

The purity and sample integrity were checked using the still fledging but powerful technique of sedimentation velocity in the analytical ultracentrifuge. An oil-turbine-driven instrument developed in Uppsala, Sweden by T. Svedberg was used at a rotor speed of 60 600 rpm. The Schlieren (refractive index gradient) optical system was employed which revealed a single hyper-sharp boundary (Figure 1). That study by R. Cecil and A.G. (Sandy) Ogston [24] in the Department of Biochemistry at the University of Oxford was also reported in the same journal as the Nottingham papers. From measurements of the sedimentation coefficient (from the sedimentation rate) and the translational diffusion coefficient (from the spreading of the sedimenting boundary), an estimate for the (weight average) molecular mass of 0.82 MDa was obtained using the sedimentation–diffusion equation of Svedberg and Pedersen after correction for non-ideality [25].

The second paper in the series, by Gulland et al. [26], involved electrometric (potentiometric) titration experiments using hydrogen and glass electrodes applied to solutions of the mildly isolated and highly purified calf-thymus DNA. The acid–base properties of preparations of DNA had been the subject of many previous investigations, but these had given conflicting results: this was largely ascribed by Gulland et al. [26] to the different degrees of degradation of the samples studied. The importance of avoiding potentially degradative and disruptive conditions in the extraction and purification procedure was stressed again: studies on poor-quality material which had been damaged or degraded were of limited use in connection with the nucleic acid structure.

## Step two: titrometric studies

Using the samples with the requisite high quality prepared by Gulland et al. [20] and starting with solutions at pH 6–7, Gulland et al. [26] followed the effects of progressive addition of either acid or alkali on, respectively, the amino groups (on the pyrimidines T and C and the purine A) or (what they believed) the enolic hydroxyl groups of T and the purine G. They observed unusual behaviour with p*K*_a_ values for the groups at more extreme pH values than expected: the groups failed to titrate as they would have done in free solution — indicating the possibility that, in the DNA molecule, the titratable groups may have been blocked from doing so by the formation of hydrogen bonds.

## Step three: viscometric and streaming birefringence measurements

The final step was made by Creeth et al. [3] who used a technique known as capillary viscometry, which provides a measure of the size of the DNA molecule in solution — and how the size can change. Earlier work had shown a reduction in viscosity (resistance to flow) on the addition of acid or alkali [27], but that reduction had been interpreted in terms of depolymerisation and had been on DNA of questionable quality (see footnote to ref. [26], and also see ref. [23]). The new study [3] was performed on the same mildly isolated high-quality and molecularly intact DNA used for the titration studies.

A Frampton [28,29] capillary viscometer was employed, which provides the advantage over conventional Ostwald capillary viscometers in being able to measure viscosity at different hydrostatic pressures. This is important for solutions of highly elongated macromolecules which could exhibit full or partial alignment under shear and hence ‘shear thinning’, a form of non-Newtonian behaviour, effects which become more marked as the pressure or shear/flow rate is increased [30].

The parameter that was measured (in a controlled temperature bath) was the relative viscosity, *η*_r_, defined by [28]

1 $$\eta_{r}=\eta /\eta_{o}$$

where *η*_r_ is the viscosity of the solution and *η*_o_ is the viscosity of the solvent. These are apparent values corresponding to particular applied pressures (or equivalent shear rates).

**Figure 2.**
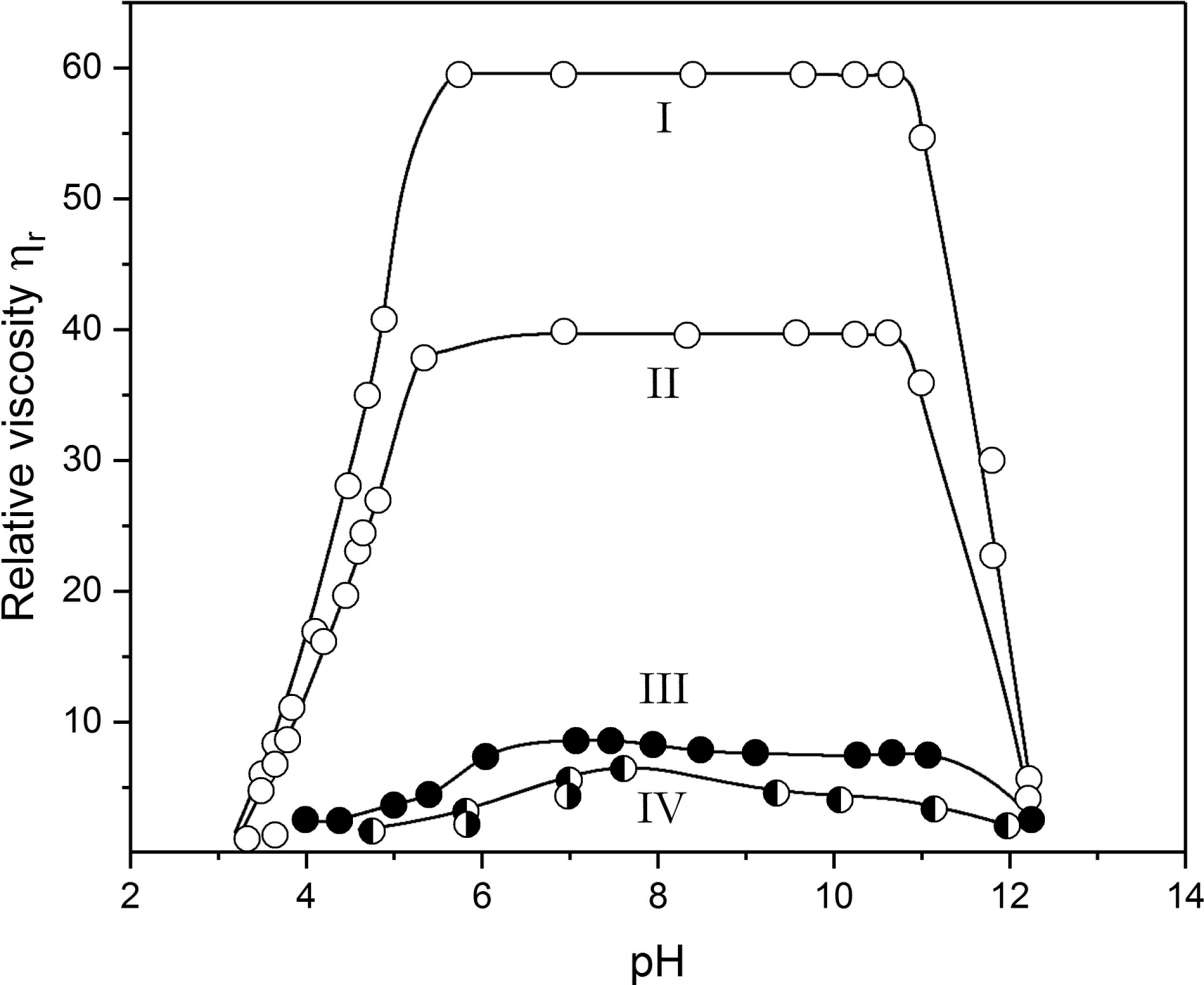
Change in relative viscosity (shown as open circles) of calf-thymus DNA isolated and purified following the Gulland et al. ‘mild procedure’ [20,23] as a function of pH in 0.01 M ionic strength solution, at a DNA concentration of 2.43 mg/ml at 25.0°C. Starting from neutral pH (6–7), either acid was added down to a pH of below 3.5, or alkali was added to a pH of over 12. Plot I, under an applied hydrostatic pressure of 3000** **dyn** **cm^−2^; Plot II, applied pressure 7000** **dyn** **cm^−2^. Also shown are the very different profiles for preparations of material after (III) alkaline pretreatment (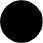) and (IV) acid pretreatment (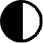), and also for a sample provided by Professor Caspersson (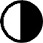). Redrawn from Creeth et al. [3].

High (apparent) relative viscosities were observed at a series of hydrostatic pressures, which increased as the pressure was dropped, confirming non-Newtonian behaviour. The dramatic way the high relative viscosity (resistance to flow) of DNA solutions dropped is shown in Figure 2 for two hydrostatic pressures at the same extremes of pH, namely below 5.6 and above 11.9 observed by electrometric titration, confirming the existence of hydrogen bonds [3]. These observations were reinforced by streaming birefringence measurements, which involved mechanical stirring of a solution placed in a small cell on the optical stage of a polarising microscope. The high birefringence caused by the alignment of long polymeric molecules at neutral pH was completely lost at the same extremes of pH observed using viscometry, again through disruption of the hydrogen bonds.

Gulland's team concluded that hydrogen bonds exist between neighbouring nucleotides, and that these are disrupted by high-acid or high-alkali conditions to give units of much smaller size [3]:

The critical pH values are coincident with those in which a liberation of amino and hydroxyl groups has been observed and it is considered that the two phenomena are related and are due to the fission of the hydrogen bonds postulated as linking the purine-pyrimidine hydroxyl groups and some of the amino-groups.

However, as to which base linked with which was not known. Furthermore — as pointed out by Booth and Hey [6] — there was also no decision at this stage that hydrogen-bonded bases on neighbouring chains led to a double-stranded structure. Initially, in the concluding remarks of the Creeth et al.'s paper, it was left open as to whether the bonding was between adjacent chains or within a single chain [3]. Indeed, it was concluded that the data could be explained by either one (or both) of the following two scenarios:

1. rupture of hydrogen bonds between adjacent chains producing units of lower molecular mass but greater asymmetry;
2. rupture of hydrogen bonds within a single chain, followed by rolling up to a more compact conformation.

In Gulland's view [19], hydrogen bonds led to aggregates or ‘micelles’ consisting of several chains, a view repeated by Jordan [31]. However, Creeth in his PhD thesis which followed soon after [4] produced his own two-chain model for DNA, which turned out to be not too far away from the true structure. Although the work was undertaken at University College Nottingham, because this was before the College received its Royal Charter, PhD's were officially external degrees of the University of London.

## The hydrogen bond discovery in the light of previous work — the issue of sample integrity

The Nottingham team led by Gulland were by no means the first to isolate and study the physico-chemical properties of DNA preparations in solution. As we have seen, by 1924 the Hammersten–Bang procedure for preparing relatively highly purified DNA had been established, and Hammersten had shown how the viscosity of DNA preparations changed in the presence of added acid. Further progress was made by Hollaender and Emmons in 1941 [32]: these researchers had also observed that preparations in water formed a heavy viscous solution, which appeared birefringent under streaming flow. They also observed ultraviolet radiation-induced depolymerisation of sodium thymonucleate. Despite reservations later expressed by Gulland et al. [26] on the quality of the DNA being used, Tennent and Vilbrandt at Wisconsin [27,33] had also characterised a DNA preparation using viscometry and analytical ultracentrifigation and estimated a chain length for the molecule of ∼5000 Å. In 1943, Villbrandt and Tennent [27] followed up and extended Hammarsten's observations on DNA titrations by using both acid and base and forward and back titrations. However, for comparable concentrations, their relative viscosities *η*_r_ were an order of magnitude lower than those of Creeth et al. [3] and instead of observing a rapid fall in *η*_r_ at pH <5.6 or >10.9, they observed a maximum in *η*_r_ and a gradual reduction in *η*_r_ as the pH was changed in either direction from neutrality: they interpreted the data in terms of a simple depolymerisation at fission points randomly along the chain. Creeth et al. [3] were able to successfully repeat the Villbrandt and Tennent profile by pretreating the original sodium salt of calf-thymus DNA with the ‘harsh’ conditions of either strong alkali at pH 12.5 or with strong acid at pH 3.5 and then precipitation of ethyl alcohol at pH 7.0. In this way, they were able to explain the anomalous results seen by these and other researchers before. The data for comparison are shown here in Figure 2. Villbrandt and Tennent were unable to see the effects that Creeth et al. [3] had seen because of the lower quality of their DNA sample (see footnote to ref. [26]).

**Figure 3.**
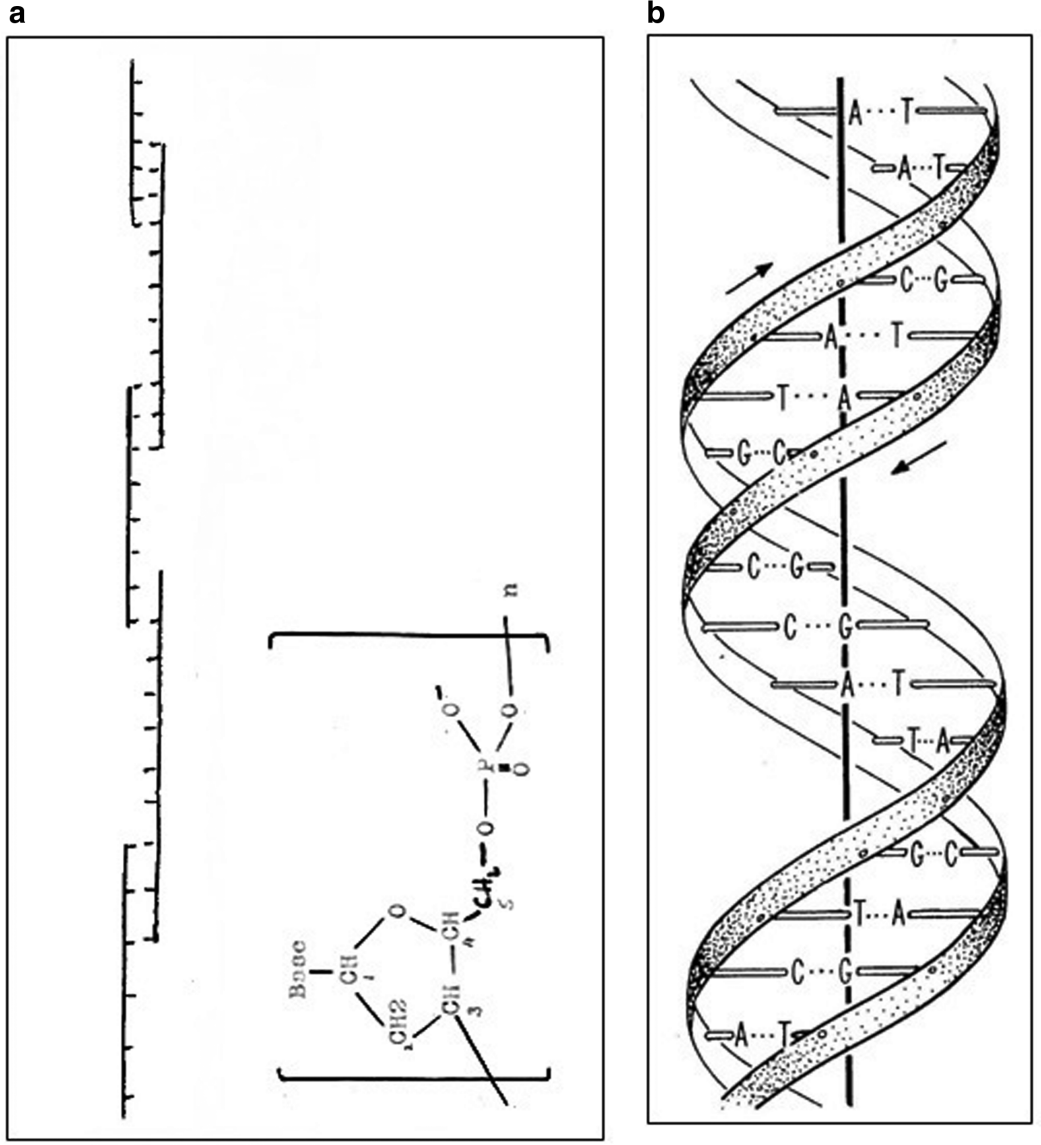
Structures for DNA. (**a**) Creeth's drawing of a possible model for the structure of DNA from his 1948 PhD Thesis (page 85) [4]: two-chain structure with sugar (deoxypentose)-phosphate backbone on the outside — represented by the two vertical lines and bases (shown as short horizontal lines) on the inside held together by hydrogen bonds. The backbone nucleotide unit (from page 79), based on his drawing from P.A. Levene, is shown on the right. (**b**) Drawing of the 1953 double-helix structure of Watson and Crick by Odile Crick [1,8], with the hydrogen bonds depicted as three dots: ^…^. Reprinted with permission of J.D. Watson. In both cases, only a small part of the molecule is shown.

## The Creeth two-chain model for the structure of DNA

**Figure 4.**
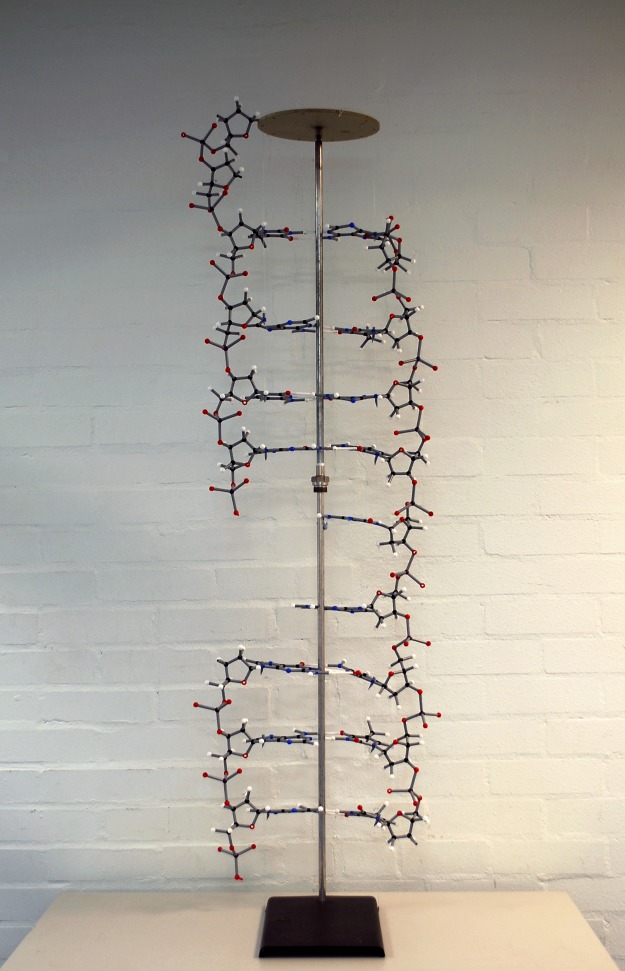
Modern ball-and-stick representation of the Creeth model. Hydrogen bonds between the bases are the white sticks/straws. How each base linked up (A, T, C and G) were not known in the Creeth model. The model is displayed in the NCMH (National Centre for Macromolecular Hydrodynamics) laboratories at Nottingham, alongside the double-helix model of Watson and Crick.

Shortly after the *Journal of the Chemical Society* publications [3,20,26], Creeth's PhD thesis appeared [4] in which, on page 85, he presented a diagram showing his two-chain model for the structure, interpreted from the viscosity, birefringence and titration results. This model is shown in Figure 3a, alongside the correct Watson–Crick two-chain double-helix model [1] for comparison (Figure 3b). A putative ball-and-stick model for the Creeth structure constructed by the authors is shown in Figure 4.

The Creeth structure from page 85 of his thesis, and shown here as Figure 3a, has two chains and is a long linear molecule with the sugar-phosphate backbone clearly and correctly on the outside of the molecule. The constituent chains are united down their common length by hydrogen bonding between facing amino and hydroxyl groups of opposite chain bases correctly on the inside of the molecule.

All these features are consistent with the correct Watson–Crick structure produced 5 years later. Creeth's model, although close, falls short on two main grounds:

1. The existence of alternating breaks with some unpaired bases in the separate chains.
2. The chains are shown as linear, not in a double helical structure.

Creeth also estimated inaccurately the number of hydrogen bonds to be a maximum of two per four phosphorus atoms. Also neither he, nor Gulland or Jordan knew of the equivalence of A with T, and C with G, or of the correct tautomeric keto forms, although Gulland was aware of the work of A.E. Mirsky, who had provided evidence that the total molecular proportions of purines = total molecular proportions of pyrimidines in earlier DNA preparations studied (see ref. [34]).

The breaks in the chain were considered consistent with the reduction in viscosity on scission of the hydrogen bonds [4]:

The action of acids and alkalis on this model is to sever the hydrogen bonds uniting the individual polynucleotide chains which are thus liberated. Being relatively small and flexible they do not interact to form the network characteristic of the micellar state, and the solution is not very viscous.

## Retrospective view of the viscosity data: there was no need to include the alternating breaks in the chains

We now include a retrospective analysis by ourselves of the original Creeth data. Evidence for long linear molecules due to non-Newtonian or shear thinning behaviour of solutions resulting from molecular alignment under shear was clearly seen from both the streaming birefringence and from the effects of hydrostatic pressure on measured relative viscosities: this is evident from Figure 2 (similar profiles are given in Figure 3 in ref. [3]) and the difference between curves I (3000 dyn cm^−2^) and II (7000 dyn cm^−2^): the birefringence largely disappears at high and low pH after the hydrogen bonds have been disrupted by titration. The non-Newtonian effects made it very difficult at the time for Gulland, Creeth or Jordan to comment further on the structure, but with more recent hydrodynamic theory (see, for example, ref. [35]) it is possible to show, in hindsight, that the viscosity behaviour is at least consistent with a halving of the molecular mass on titration.

**Figure 5.**
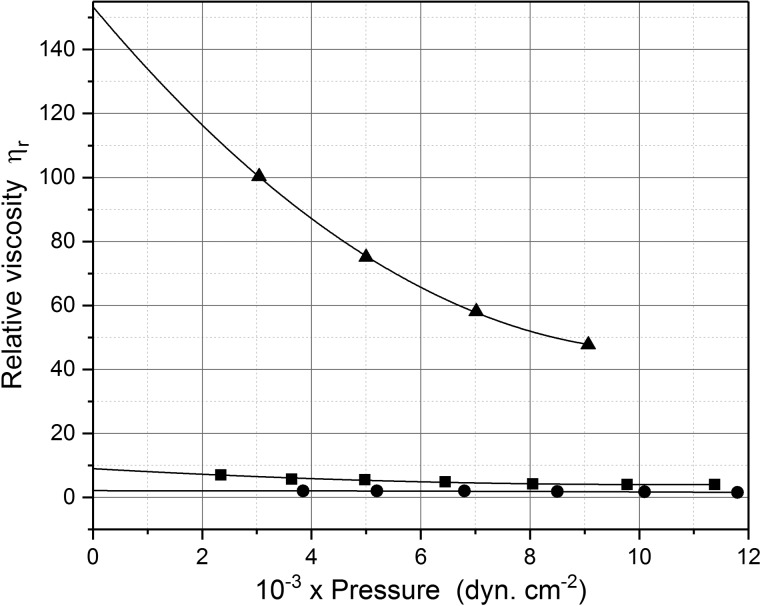
Extrapolation of relative viscosities for calf-thymus DNA solutions at neutral pH to zero hydrostatic pressure. Triangles: original solution at 2.43 mg/ml (from Figure 4 curve IX of [3]): *η*_r_ ∼ 150. Circles: original solution at 0.12 mg/ml (from Graph 3/2 curve V of [4]): *η*_r_ ∼ 2. Squares: solution at 2.43 mg/ml shortly (25 min) after alkaline treatment (from Figure 4 curve V of [3]): *η*_r_ ∼ 10.

Firstly, we utilise a dataset of relative viscosity *η*_r_ versus hydrostatic pressure under neutral pH conditions (for a concentration of 0.243% or 2.43 × 10^−3^ g/ml) in Figure 4 of ref. [3]. Extrapolation of this dataset to zero pressure yields a value for the *η*_r_ which is close to Newtonian: a value of *η*_r_ ∼ 150 is obtained (Figure 5 and Table 1).

**Table 1:** Zero hydrostatic pressure relative *η*_r_ and approximate intrinsic [*η*] viscosities

Conditions	*η*_r_	[*η*] (ml/g)
Neutral pH, 2.43 mg/ml	150	∼7000
Neutral pH, 0.12 mg/ml	2	∼7000
After alkaline treatment, 2.43 mg/ml	10	1500

We can obtain an estimate for [*η*] using the approximate relation of Solomon and Ciuta [36]:

2 $$[\eta ]=\{1/c\}\cdot [2(\eta_{r}-1)-2\ln\eta_{r}{]}^{0.5}$$

which negates the need for a concentration extrapolation. This relation — not available in 1947 — yields a value for [*η*] of ∼7000 ml/g. As a check we can use a dataset obtained at a lower concentration (0.12 × 10^−3^ g/ml) from Graph 3/2 of Creeth's PhD thesis [4]. From the much smaller variation of *η*_r_ with pressure, a zero pressure value of *η*_r _∼ 2 is obtained (Figure 5), reassuringly leading to a similar estimate for [*η*] of ∼7000 ml/g. Finally, also from Figure 4 of ref. [3], we can estimate a zero pressure extrapolated value of *η*_r_ of ∼10 at 2.43 mg/ml [yielding a drop in the intrinsic viscosity to ∼1500 ml/g (Figure 5)].

Secondly, we can use the Mark–Houwink–Kuhn–Sakurada (MHKS) relation (see ref. [35] and references cited therein) linking intrinsic viscosity for a homologous series of polymers to see if a dissociation of the double-stranded model to give two complete rather than two broken chains is consistent with the data. The MHKS relation is

3 $$[\eta ]=\kappa_{\eta }\cdot M^{a}$$

where *κ_η_* is a constant for a homologous polymer series and *a* is the MHKS scaling coefficient. For (non-draining) spheres *a* = 0, for random coils *a* = 0.5–0.7 and for rods *a *= 1.8 (see refs [35,37]).

For a rod-shaped molecule, the expected reduction in intrinsic viscosity corresponding to a halving of the molar mass as the two chains come apart would from eqn (3) lead to a maximum reduction of 3.5× (corresponding to *a* = 1.8), bringing the intrinsic viscosity down from 7000 to ∼2000 ml/g. Bearing in mind that the single-chain molecules will be considerably more flexible than their double-chained counterparts — and hence their viscosities will be lower to a certain degree, this plus a halving in molecular mass through hydrogen bond disruption would not be an implausible explanation of the results. We stress that this is our modern calculation, not a review of Creeth's from his thesis.

Other recent work has shown that single-chain nucleic acids are much more flexible — lower persistence lengths of ∼2 nm [30] than the more rigid double helical structure of ∼40 nm [38].

So, the two-chain hydrogen-bonded model for DNA given in Creeth's PhD thesis could, on the basis of our calculation, have accounted for the viscosity data available at the time and without the need for alternating breaks in the chains. The second scenario — that the action of titrating with acid or alkali could also lead to a fission of intra-chain hydrogen bonds between bases along the same chain — is also consistent with the drop in viscosity and loss of birefringence, and Creeth repeats this in his thesis. Although the molecular mass would then remain the same, the removal of the bonds could encourage the chain to take a more flexible, less-extended conformation resulting in a lowering of viscosity and loss of birefringence.

In his thesis [4], Creeth concludes (page 91) ‘the macromolecular structure of sodium deoxyribonucleate isolated by mild methods is that of a very long, very asymmetric rigid particle. Further, it has been shown that this condition is in all probability due to the presence of hydrogen bonds’.

Besides the breaks, also missing from the Creeth two-chain model is, of course, the double helix and the correct pairing of the bases. In 1952, Watson and Crick were given access to the high-resolution X-ray diffraction image — the famous ‘Photo 51’ of Rosalind Franklin and R.G. Gosling (see ref. [8]), with the characteristic cross and intensity pattern of a double helix with ‘space group C2’ — two chains running antiparallel with each other. This image was generated from ‘Signer’ DNA [16], of similar high purity to that from the Nottingham group. Florence Bell and W.T. Astbury did not have access to this high-quality DNA when they published their diffraction images in 1939 [17,18]. It is interesting to speculate that if the Nottingham team had made available their high-purity DNA, could the Leeds team have produced images of the same quality as Rosalind Franklin's. In 1946, Gulland and Astbury both attended the same meeting — a meeting of the Society of Experimental Biology in Cambridge, and both contributed to the Proceedings published a year later [23,34,39] — this may have been an opportunity lost.

Later, the Leeds team were able to produce a high enough quality image: in 1951, E. Beighton, a researcher in Astbury's laboratory, produced a clear diffraction pattern with the characteristic helical cross (see ref. [40]), almost a year before the Kings College London Group produced ‘Photo 51’. But, by then, it was too late as the Nottingham team had all but gone: Creeth had finished his PhD and had become a research Fellow at the Courtauld Institute in London before moving to the Department of Physical Chemistry in Wisconsin [41]. His supervisor Doj Jordan moved to take up an appointment at the University of Adelaide, and tragically Masson Gulland was killed in a train crash in October 1947 [6], not long after the publication of the hydrogen bond finding.

**Figure 6.**
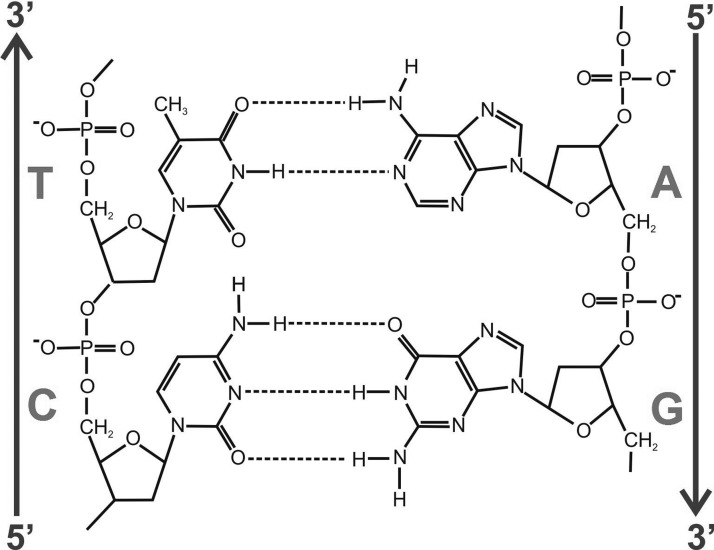
Section of the Watson–Crick DNA structure showing hydrogen bonds connecting the pyrimidine bases thymine (T) and cytosine (C) with, respectively, the purine bases adenine (A) and guanine (G). The precise hydrogen bond link between the bases made by Watson and Crick followed from the Chargraff base-pair rules and from J. O'Donohue's identification of the correct (*keto*) tautomeric form. Adapted with permission from Booth and Hey [6]. Copyright (1996) American Chemical Society.

The Nottingham team also would not have had access to the findings of Chargaff et al. on the equivalence of the bases — not published until 1950. And finally, the Nottingham team were working on the assumption that the thymine and guanine bases were in the enolic hydroxyl tautomeric form (–COH=N–), a commonly held view at that time, and included in J.N. Davidson's classic text [42] used by Watson. As famously recalled by J.D. Watson in his book [8], he and Crick had been struggling to get the T–A and C–G base pairs to fit into the helical structure. J. Donohue, a Guggenheim Fellow sharing an office with them at the Cavendish — and an expert on tautometric forms — was able to point out to Watson that Davidson was wrong, and the T and G bases were primarily in the keto form (–CO–NH–) at physiological pH's [8]. This enabled Watson and Crick to complete their model, with the bases hydrogen bonded in their correct tautomeric forms (Figure 6), allowing a regular stacking of bases within the antiparallel double helical frame of the sugar-phosphate backbone and at the correct spacing.

## Concluding remarks

To the modern-day molecular biologist, it is not hard to recognise that the diagram drawn by Creeth [4] in his 1948 PhD thesis (Figure 3a) bears similarities to sketches that might be drawn today for the design of PCR (polymerase chain reaction) experiments using multiple annealing primers. It also resembles current textbook diagrams depicting mechanisms by which some viruses integrate into host chromosomes during their replication cycles using staggered cuts in the duplex and the resulting production of short single-stranded segments (e.g. bacteriophage Mu — see ref. [43]) or indeed the mechanisms by which some viruses replicate their nucleic acids (e.g. double-stranded DNA synthesis generated from retroviral single-stranded RNA, [44]). Of course, in 1947, the processes of DNA replication (or indeed PCR amplification) were not yet discovered; the correct semi-conservative replication model of Watson and Crick was not proposed until 1953 [45] and not confirmed until 1958 using analytical ultracentrifugation [46]. Nor was it yet known in 1947 what the mechanism of heredity was. So, although Creeth's model is a depiction of DNA structure alone, he could not have realised how it resembles what we now understand about steps in some replicative mechanisms for the molecule of life that he was working on.

**Figure 7.**
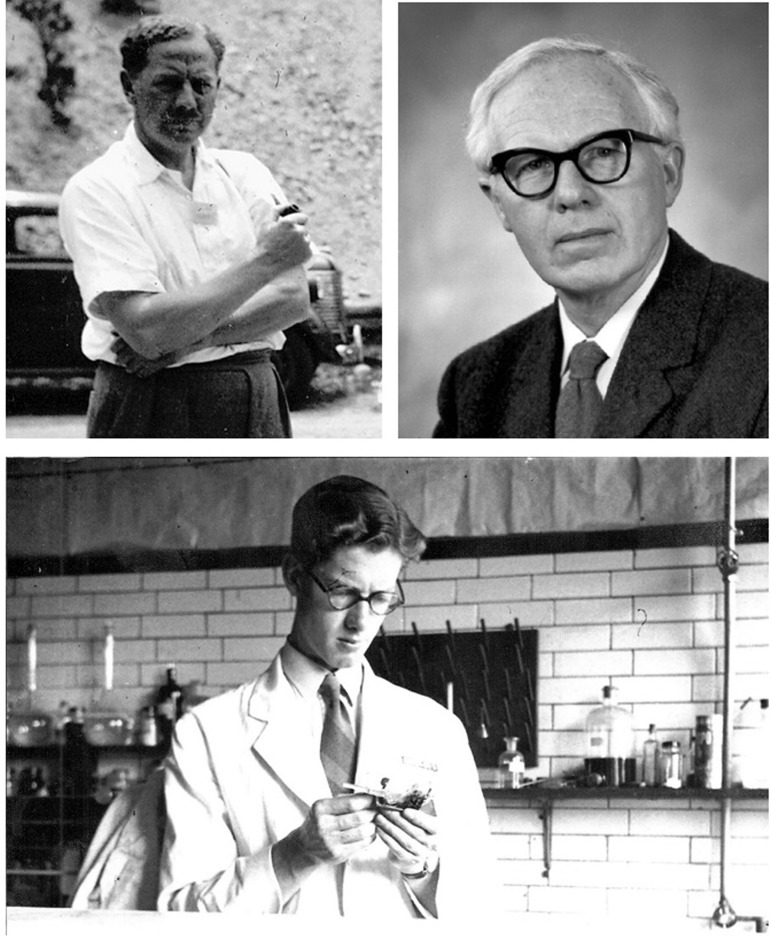
Gulland, Jordan and Creeth. Top left: J.M. Gulland, from a photograph taken at the 1947 Symposia Nucleic Acids and Nucleoproteins. Courtesy of Cold Spring Harbor Laboratory Library and Archives. Top right: D.O. Jordan. Bottom: J.M. Creeth, photograph taken ca. 1947.

In Mike Creeth's own words, looking back after his retirement [47]: ‘In hindsight, we had been given not just a glimpse, but a good view of that particular bonding that is nothing less than the key to life on this planet.’ He, and his distinguished supervisors Gulland and Jordan (Figure 7), and fellow students Threlfall and Taylor, helped pave the way for the final discovery by Watson and Crick of the structure of the macromolecule that is the key to life, but, characteristically, was too modest to say that.

## Abbreviations

DNA: deoxyribonucleic acid
PCR: polymerase chain reaction
RNA: ribonucleic acid
